# Trends in *Escherichia coli* and *Klebsiella pneumoniae* Urinary Tract Infections and Antibiotic Resistance over a 5-Year Period in Southeastern Gabon

**DOI:** 10.3390/antibiotics14010014

**Published:** 2024-12-28

**Authors:** Yann Mouanga-Ndzime, Cyrille Bisseye, Neil-Michel Longo-Pendy, Michelle Bignoumba, Anicet-Clotaire Dikoumba, Richard Onanga

**Affiliations:** 1 Medical Research and Analysis Unit, Bacteriology Laboratory, Interdisciplinary Centre for Medical Research of Franceville, Franceville P.O. Box 769, Gabon; bignoumba_michelle@yahoo.fr (M.B.); dikoumba@hotmail.com (A.-C.D.); onangar@yahoo.com (R.O.); 2 Department of Biology, Faculty of Sciences, University of Science and Technology of Masuku, Franceville P.O. Box 943, Gabon; 3 Research Unit for the Ecology of Health, Interdisciplinary Centre for Medical Research of Franceville, Franceville P.O. Box 769, Gabon; pendyneils@yahoo.fr

**Keywords:** UTIs, *Escherichia coli*, *Klebsiella pneumoniae*, sociodemographic and climatic factors, antibiotic resistance

## Abstract

**Background:** Urinary tract infections (UTIs) are a substantial global health concern, exacerbated by the widespread use of antibiotics and leading to the development of multidrug-resistant strains. The aim of this study was to analyze the temporal patterns of *Escherichia coli* and *Klebsiella pneumoniae* UTIs and antibiotic resistance, taking into account various sociodemographic, clinical, and climatic factors within the study population. **Methods:** A total of 3026 urine samples from patients of all ages were analyzed over a period of five years by standard microbiological methods. Climatic data for the study area were also collected. Univariate and multivariate logistic regression analyses were performed to measure the impact of sociodemographic, clinical and climatic parameters on the occurrence of UTIs. **Results:** The study showed a 31.4% prevalence of UTIs among the population. Notably, there was a significant increase in pyelonephritis between 2019 and 2023 (*p* < 0.01). Furthermore, a significant association was found between cystitis and the long dry season, as well as the short rainy season. Furthermore, *Escherichia coli* and *Klebsiella pneumoniae* exhibited resistance to beta-lactams, quinolones, and co-trimoxazole. The resistance of *Escherichia coli* isolated from cystitis to nitrofurantoin showed a significant increase over the years (*p* < 0.04). Principal component analysis (PCA) suggested that humidity may play a role in the emergence of multidrug-resistant strains of *Escherichia coli* and *Klebsiella pneumoniae*. **Conclusions:** UTIs show variability according to various sociodemographic, clinical, and climatic factors, with a higher risk of complications seen in individuals aged ≤ 17 years. It is important to note that cases of pyelonephritis have been increasing over time, with a noticeable seasonal variation. This study suggests that humidity may play a role in promoting antibiotic multidrug resistance in *Escherichia coli* and *Klebsiella pneumoniae*.

## 1. Introduction

Urinary tract infections (UTIs) are one of the most common types of infections globally, but there is a lack of complete data on long-term trends. In 2019, it was estimated that there were 404.61 million cases of UTIs worldwide, resulting in around 236,790 deaths [1]. UTIs are traditionally categorized into three distinct groups: asymptomatic bacteriuria, acute cystitis (bladder infection), and acute pyelonephritis (kidney infection). This classification is crucial for the accurate diagnosis and effective treatment of UTIs [2]. The currently recommended empirical antimicrobial regimen for treating acute uncomplicated cystitis includes antibiotics such as amoxicillin-clavulanic acid, cefixime, fluoroquinolones (ciprofloxacin or ofloxacin), fosfomycin, and nitrofurantoin. Trimethoprim/sulfamethoxazole could be considered a first-choice drug but only if local resistance to *Escherichia coli* (*E. coli*) does not exceed 20 percent. For the treatment of acute uncomplicated pyelonephritis, it is advised to use cefotaxime, ceftriaxone, and fluoroquinolones [3]. However, the ongoing development of resistance, especially to fluoroquinolones and cephalosporins, highlights the importance of regularly updating these recommendations at the local level to prioritize antibiotics that have minimal impact on the intestinal microbiota.

The economic burden of UTIs is compounded by the rising prevalence of antibiotic resistance, which results in more intricate and costly therapeutic interventions. In the community setting, UTIs represent the second most prevalent infectious presentation in primary care, with an estimated economic burden of over USD 6 billion annually on a global scale [4].

These infections are a major public health concern for people of all ages, affecting both infants and the elderly, regardless of gender [5]. UTIs have been identified as a significant contributor to morbidity and healthcare expenditure, particularly in low- and middle-income countries (LMICs) [6]. The most prevalent pathogens responsible for UTIs are Gram-negative bacteria belonging to the Enterobacteriales group. These pathogens encompass uropathogenic *E. coli* and the *Klebsiella pneumoniae* (*K. pneumoniae*) *complex* [7].

The latest report from the Center for Disease Control (CDC) estimates that antibiotic-resistant infections have an annual impact of 2.8 million cases of infection and 35,000 deaths in the United States. The majority of deaths were attributed to six AMR pathogens: *E. coli*, *Staphylococcus aureus* (*S. aureus*), *K. pneumoniae*, *Streptococcus pneumoniae* (*S. pneumoniae*), *Acinetobacter baumannii* (*A. baumannii*), and *Pseudomonas aeruginosa* (*P. aeruginosa*) [8].

The emergence of drug-resistant bacteria in the community represents a significant and imminent threat to public health, with the potential to increase morbidity, mortality, healthcare costs, and the utilization of antibiotics [9,10]. In 2020, a study of urinary tract infections caused by *E. coli* revealed that one in five cases showed reduced susceptibility to standard antibiotics, including ampicillin, co-trimoxazole, and fluoroquinolones. This situation presents a significant challenge to the effective treatment of these common infections [11]. Moreover, a recent study has demonstrated that the prevalence of multidrug-resistant (MDR) isolates among patients with community-acquired UTIs exhibits considerable variation across African countries [12].

Two recent studies conducted in Gabon’s southeastern region have revealed that *E. coli* and *K. pneumoniae* are the most prevalent pathogens in both pediatric urinary tract infections and those occurring in the adult population [13,14]. These findings are consistent with global data indicating that *E. coli* and *K. pneumoniae* are the most prevalent uropathogens. However, the specific patterns of resistance observed in Gabon differ from those reported in high-income countries, underscoring the importance of regional studies for understanding the local dynamics of antimicrobial resistance (AMR). Despite the growing global recognition of AMR as a major health threat, data from LMICs like Gabon remain limited. The paucity of comprehensive, long-term data on the prevalence and resistance patterns of urinary tract pathogens in Gabon impedes the development of effective national treatment guidelines. It is therefore imperative that localized research be conducted in order to bridge this knowledge gap and to ensure that public health policies reflect the realities on the ground. In the absence of robust local data, global recommendations may prove inadequate in addressing the specific needs of regions such as Gabon, where healthcare infrastructure, antibiotic use and environmental factors differ significantly from those in high-income settings.

Furthermore, the tropical climate of Gabon introduces additional variables, including seasonal fluctuations in temperature and humidity, which can influence the occurrence and spread of UTIs, as well as patterns of resistance. Emerging evidence suggests that infections caused by Gram-negative bacteria exhibit seasonal trends, with peaks typically occurring during the warmer summer months [15,16,17].

Understanding how these factors interact with AMR trends is critical not only for guiding local treatment protocols but also for contributing valuable insights to the global fight against antibiotic resistance. This study aims to provide a deeper understanding of *E. coli* and *K. pneumoniae* UTIs trends and resistance patterns in the Southeast of Gabon, offering data that can inform both local healthcare strategies and international discussions on AMR.

## 2. Results

### 2.1. General Patient Information in the Study

A total of 3026 urine samples were collected over a 5-year period. Women were predominantly represented in the study population, comprising 58.5% (1769/3026) of it. The male/female sex ratio was 0.71, while the mean age of patients was 23.37 ± 19.89 years. The median age of pediatric participants was 2 years (interquartile range [IQR], 0.75–7 years), while the median age for adults was 33 years (IQR, 27–39 years). In the elderly population, the median age was 57 years (IQR, 53–65 years).

Patients in the 18–49 age group were the most represented, comprising 45% (1363/3026) of the study population, and the majority of them resided in urban areas (71.4%), with an urban/rural ratio of 2.5 (Table 1). A total of 949 cases of UTIs were documented among the 3026 individuals included in the study, representing an overall prevalence of 31.4. The prevalence of UTIs was found to be higher in women compared to men (33.5% vs. 28.3%, *p* = 0.002). When analyzing age groups, individuals aged ≤ 17 years and those aged 50 years and older exhibited higher infection rates at 34.2% and 33.0%, respectively, while the 18–49 age group showed the lowest prevalence of UTIs, at 28.2% (*p* = 0.003). Furthermore, urban residents had a higher prevalence of UTIs compared to those in rural areas (32.5% vs. 28.4%, *p* = 0.03). Interestingly, the highest prevalence of UTIs was observed in 2022 at 39.6%, with a significant decrease noted in 2020 at 21.0% (*p* < 0.0001). Although seasonal variations were observed, they were not found to be statistically significant (Table 1).

### 2.2. A Five-Year Analysis of the Incidence of Urinary Tract Infections

**Figure 1:**
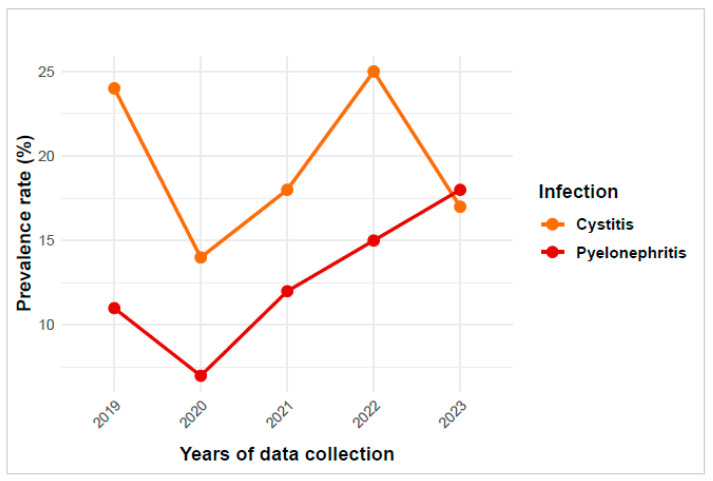
Trend in urinary tract infections over 5 years. This plot displays the prevalence rates of cystitis and pyelonephritis across the annual data over five years.

The study showed that 31.4% (949/3026) of the samples tested were positive for a UTI. Cystitis constituted 62% of UTIs (589/949), while pyelonephritis accounted for 38% (360/949). The prevalence of cystitis was highest in 2022 (25%), while that of pyelonephritis was higher in 2023 (18%) (Figure 1). A statistically significant reduction in the incidence of cystitis was observed when comparing the data from 2023 to those from 2019 (*p* = 0.02). No such difference was observed when comparing the years 2020, 2021, and 2022 with 2019 (Table 2). As shown in Table 2, the incidence of pyelonephritis was markedly elevated in 2023 in comparison to that in 2019.

**Figure 2:**
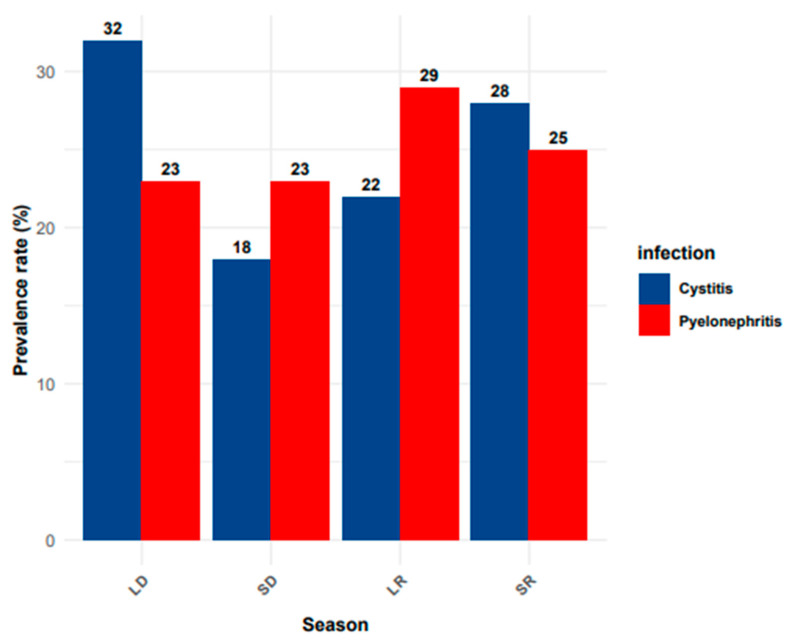
Seasonal prevalence of cystitis and pyelonephritis. The bar chart displays the prevalence rates (%) of cystitis and pyelonephritis across different seasons. The seasons are categorized as LD (long dry season), SD (short dry season), LR (long rainy season), and SR (short rainy season).

In analyzing the distribution of cystitis and pyelonephritis according to sociodemographic parameters, it was found that there was no correlation with the age of the patients, as shown in Table 2. However, a significantly higher prevalence of cystitis was observed in female patients compared to male patients (*p* < 0.001). No significant discrepancy was identified between rural and urban regions with respect to the prevalence of both types of UTIs (Table 2). Cystitis was the only condition found to be significantly associated with seasonality (*p* < 0.0001). A significant association was observed between cystitis and the long dry season in comparison to the short dry season (*p* < 0.0001) and the long rainy season (*p* < 0.001). Furthermore, a significant association was found between cystitis and the short rainy season in comparison to the short dry season (*p* < 0.001) (Figure 2). No significant association was observed between pyelonephritis and seasonality. However, it was noted that this condition was more prevalent among patients during the long rainy season (Figure 2).

### 2.3. Distribution of UTIs According Clinical Categories

UTIs were classified into two categories: uncomplicated infections and infections with risk of complications. A significant association was observed between cystitis (*p* < 0.0001) and pyelonephritis (*p* < 0.0001) at risk of complications and the age group ≤ 17 years (Table 3). No difference was observed between uncomplicated cystitis and cystitis at risk of complication in both urban and rural areas. However, in urban areas, the prevalence of pyelonephritis at risk of complications was significantly higher compared to uncomplicated pyelonephritis (*p* < 0.01) (Table 3). *E. coli* isolates was found to be associated with both uncomplicated cystitis (*p* < 0.0001) and pyelonephritis (*p* = 0.02). No significant associations were observed between these factors and *K. pneumoniae* isolates (Table 3).

### 2.4. Distribution of E. coli and K. pneumoniae According to Sociodemographic, Seasonal, and Temporal Parameters over a Five-Year Period

**Figure 3:**
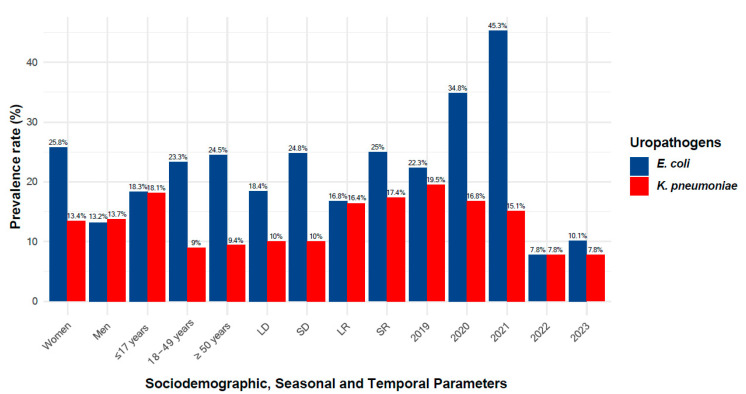
Prevalence trends of *E. coli* and *K. pneumoniae* isolates across sociodemographic, seasonal and temporal parameters. This plot illustrates the prevalence rates of *E. coli* and *K. pneumoniae* isolates across various sociodemographic parameters (such as gender and age groups), seasonal factors, and annual data over five years.

A total of 200 strains of *E. coli* were isolated from patients’ urine. A significant association was observed between *E. coli* and UTIs in women compared to men (25.8% vs. 13.2%, *p* < 0.0001). Similarly, there was a significant increase in the prevalence of *E. coli* infections in the years 2020 (*p* < 0.01) and 2021 (*p* < 0.001), as compared to other years (Figure 3). *E. coli* was associated neither with patients’ age nor with seasons.

In this study, a total of 129 strains of *K. pneumoniae* were isolated from urinary tract infections. The prevalence of *K. pneumoniae* was significantly higher in patients aged ≤17 years compared to that in other age groups (*p* < 0.001) (Figure 3). Additionally, there was a significantly higher incidence of *K. pneumoniae* in 2019 compared to 2022 and 2023 (19.5% vs. 7.8%, *p* < 0.01) (Figure 3). The distribution of *K. pneumoniae* was not associated with patient sex or seasonality.

### 2.5. Antibiotic Resistance of E. coli and K. pneumoniae Strains Isolated from Patients with Cystitis and Pyelonephritis

**Figure 4:**
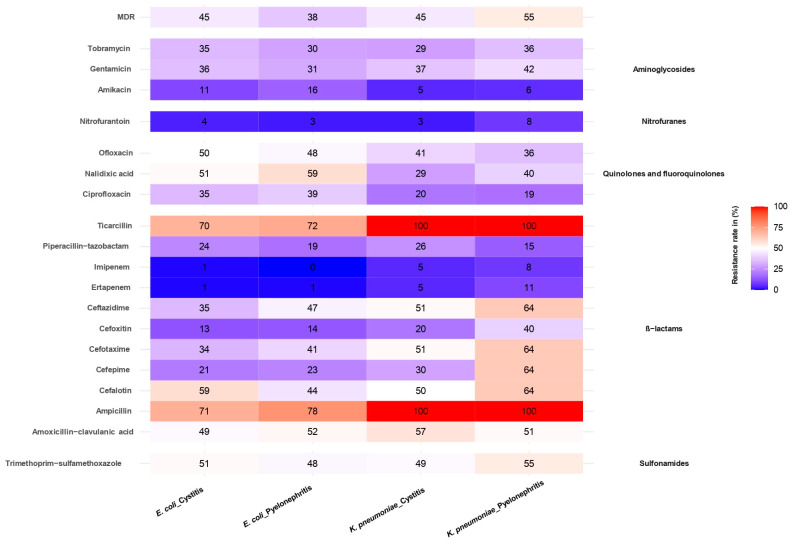
Antibiotic resistance profiles of *E. coli* and *K. pneumoniae* isolated from cystitis and pyelonephritis. This heatmap shows antibiotic resistance rates (%) for *E. coli* and *K. pneumoniae* isolates from cystitis and pyelonephritis. Resistance is presented for various antibiotic classes, with red indicating high resistance (up to 100%) and blue representing low resistance (close to 0%). Multidrug resistance (MDR) is displayed at the top.

Both *E. coli* and *K. pneumoniae* showed a high frequency of resistance to a range of antibiotic classes. Of the 136 *E. coli* strains isolated from cystitis cases, the highest resistance rates were observed for the following antibiotics: ampicillin (71%), ticarcillin (70%), cephalothin (59%), nalidixic acid (51%), trimethoprim-sulfamethoxazole (51%), ofloxacin (50%), and amoxicillin-clavulanic acid (49%) (Figure 4). On the other hand, low levels of *E. coli* resistance were reported for the following antibiotics: ertapenem (1%), imipenem (1%), nitrofurantoin (4%) and amikacin (11%) (Figure 4). Multidrug resistance to antibiotics was observed in 45% of *E. coli* strains isolated from cases of cystitis.

In this study, a total of 64 strains of *E. coli* were isolated from cases with pyelonephritis. The highest resistance rates were obtained for the following antibiotics: ampicillin (78%), ticarcillin (72%), nalidixic acid (59%), amoxicillin-clavulanic acid (52%), ofloxacin (48%), trimethoprim-sulphamethoxazole (48%), and ceftazidime (47%). The lowest resistance rates were observed for ertapenem (1%) and nitrofurantoin (3%), while all strains were sensitive to imipenem (Figure 4). Multidrug resistance was observed in 38% of the *E. coli* strains isolated from cases with pyelonephritis (Figure 4). In addition, no statistically significant differences were observed when comparing the antibiotic resistance of *E. coli* strains isolated from cystitis to those isolated from pyelonephritis. Of the 76 *K. pneumoniae* strains isolated from cases of cystitis, the highest-resistance rates were observed for the following antibiotics: amoxicillin-clavulanic acid (57%), cefotaxime (51%), ceftazidime (51%) and trimethoprim-sulfamethoxazole (49%) (Figure 4). The lowest-resistance rates were observed for ertapenem (5%), imipenem (5%), amikacin (5%) and nitrofurantoin (3%) (Figure 4). A total of 45% of the *K. pneumoniae* isolates were multidrug-resistant strains associated with cystitis. With regard to the 53 *K. pneumoniae* strains isolated from patients with pyelonephritis, the highest-resistance rates were observed for the following antibiotics: cephalothin (64%), cefotaxime (64%), ceftazidime (64%), cefepime (64%), trimethoprim-sulfamethoxazole (55%) and amoxicillin-clavulanic acid (51%). The lowest resistance levels were observed for imipenem (8%), nitrofurantoin (8%) and amikacin (6%) (Figure 4). Multidrug resistance was identified in 55% of *K. pneumoniae* strains isolated from pyelonephritis (Figure 4). Furthermore, the rates of resistance to the antibiotics cefoxitin (*p* = 0.02) and cefepime (*p* < 0.001) were found to be significantly associated with *K. pneumoniae* strains isolated from pyelonephritis in comparison to those isolated from cystitis.

### 2.6. Principal Component Analysis (PCA) of Climatic Factors Associated with Uropathogen Multidrug Resistance

**Figure 5:**
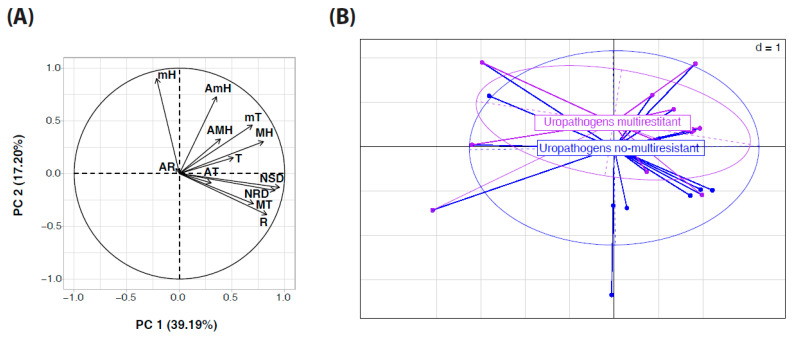
Principal component analysis of multidrug resistance and climatic factors. This figure illustrates the association between multidrug resistance and various climatic factors, including temperature, humidity, precipitation rate, number of rainy days, and number of stormy days.

A principal component analysis (PCA) was conducted to evaluate the associations between multidrug resistance in uropathogenic strains (*E. coli* and *K. pneumoniae*) and various climatic factors. The first two principal components (PC1 and PC2) collectively accounted for 56.47% of the total variance observed in the dataset, with PC1 accounting for 39.19% and PC2 accounting for 17.28% of the total variance (Figure 5A).

Figure 5A illustrates the projection of climatic factors onto the initial two principal axes. The various forms of humidity (monthly maximum humidity [MH], monthly minimum humidity [mH], annual maximum humidity [AMH], and annual minimum humidity [AmH]) demonstrated a robust correlation with PC1, suggesting a significant influence on the differentiation of the samples. In contrast, temperature variables (monthly maximum temperature [MT], monthly minimum temperature [mT], and annual average temperature [AT]) were primarily correlated with PC2. The precipitation-related factors, namely the annual number of precipitation days (R), the annual number of thunderstorm days (NSD), and the annual number of rainy days (NRD), exerted a more moderate influence on the explained variance.

These findings indicate that humidity, whether measured on a seasonal or annual basis, is the most discriminating climatic factor in distinguishing between multidrug-resistant and non-multidrug-resistant strains.

Figure 5B depicts the distribution of multidrug-resistant and non-multidrug-resistant strains within the factor space, accompanied by 95% confidence ellipses for each group. The occurrence of multidrug-resistant strains is significantly correlated with elevated humidity levels, as evidenced by their clustering in regions where the vectors representing different forms of humidity (mH, MH, AmH, AMH) are concentrated. In contrast, non-multidrug-resistant strains display a more extensive distribution, indicating a comparatively weaker correlation with the analyzed climatic factors.

These findings highlight the pivotal role of humidity as a principal environmental factor influencing the emergence and dissemination of multidrug resistance among uropathogens in this region.

### 2.7. Temporal Distribution of Antibiotic Resistance of Interest in E. coli and K. pneumoniae

**Figure 6:**
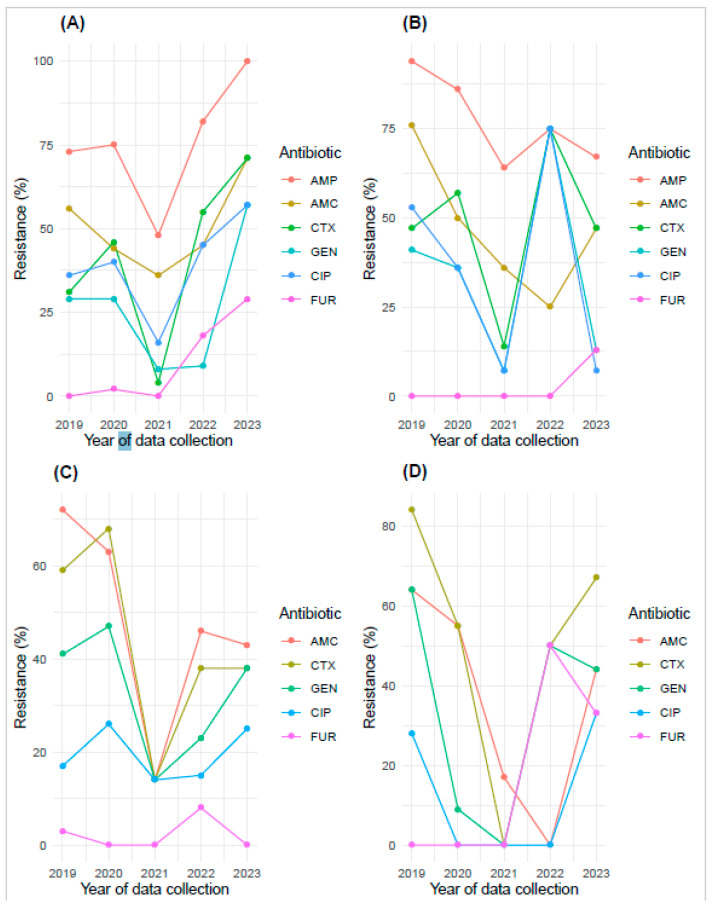
Trends in antibiotic resistance of interest in *E. coli* and *K. pneumoniae* isolated from UTIs over five years. This figure displays the trends in antibiotic resistance of *E. coli* and *K. pneumoniae* isolates from cystitis cases (**A**,**C**) and pyelonephritis cases (**B**,**D**) over the past five years, respectively. The data include resistance percentages for several antibiotics, showing how resistance levels have changed annually.

The evolution of antibiotic resistance over time was determined using logistic regression (Supplementary Tables S1–S4). The resistance of *E. coli* strains isolated from cystitis to nitrofurantoin showed a significant increase over time (*p* < 0.04) (Figure 6A and Supplementary Table S1). A significant increase in antibiotic resistance was observed across all tested drugs from 2021 to 2023 in *E. coli* strains isolated from cases of cystitis (Figure 6A). With regard to other antibiotics such as ampicillin, amoxicillin-clavulanic acid, cefotaxime, gentamicin, and ciprofloxacin, the *E. coli* and *K. pneumoniae* strains isolated from cystitis and pyelonephritis exhibited non-significant variable resistance rates over time (Figure 6B–D; Supplementary Tables S1–S4).

## 3. Discussion

The objective of this study was to ascertain the temporal evolution of UTIs and antibiotic resistance in relation to the socio-clinical factors of the patients and the climatic factors of the study area. The prevalence of UTIs observed in this study is in agreement with that reported in a previous study carried out in the southeast of Gabon [13]. However, this differs from the reported prevalence of UTIs of 59.8% in Cameroon [18]. This study revealed a notable surge in the frequency of pyelonephritis cases in 2023 when compared to data from 2019. This result is in accordance with the conclusions of a recent report that demonstrates an upward trend in the incidence of UTIs globally, including pyelonephritis [1,19]. The increase in pyelonephritis cases may be attributed to a number of factors, including the indiscriminate prescription of antibiotics, particularly in children, or the emergence of antibiotic-resistant bacterial strains. This rise in pyelonephritis cases merits particular attention, as it may signal an increased requirement for novel and more efficient prevention and management strategies for UTIs. Furthermore, the study showed that the prevalence of cystitis was significantly higher in women than in men. This result is in accordance with the findings of previous studies [20,21,22]. The observed difference in the prevalence of cystitis between women and men can be attributed to several factors. On the one hand, the length of the urethra in men, the proximity of the urethral, anal, vaginal orifices, and poor hygienic practices in women may contribute to the observed difference. On the other hand, pregnancies have been shown to induce lowered immune responses that favor the development of microbial agents in pregnant women [23,24].

The results of this study demonstrated a statistically significant correlation between the prevalence of cystitis and seasonal patterns. A notable increase in the incidence of UTIs was observed during the long dry season and the short rainy season. This finding is consistent with that of previous studies conducted in southeastern Gabon, as well as with research from the United States, which revealed a seasonal increase in hospital admissions for UTIs [13,25]. The observed increase in cystitis cases may be attributed to seasonal variations in water consumption, as well as the intricate interplay between host-related and environmental factors, and the environmental dynamics of pathogens.

Interestingly, however, other studies globally have documented a seasonal increase in UTIs during the summer months [26,27], particularly infections caused by Gram-negative bacteria, which show higher prevalence during warmer periods [16,28,29]. This apparent contradiction to our findings suggests that local environmental and behavioral factors may exert a stronger influence in determining the epidemiological patterns of UTIs in different regions. In the Gabonese context, the data indicate a higher incidence of cystitis during the colder seasons, specifically the long dry season and the short rainy season, which contrasts the globally observed summer peak.

This anomaly can potentially be explained by region-specific behavioral and physiological factors. One plausible explanation is the increased frequency of sexual activity during colder months, a well-recognized risk factor for the onset of UTIs, particularly in young adults. Sexual intercourse is known to facilitate bacterial colonization of the urinary tract, making it a primary contributor to the occurrence of cystitis [3]. Furthermore, the colder seasons are often accompanied by a reduction in fluid intake, which is likely due to decreased thirst in response to lower temperatures. Insufficient hydration represents a critical factor in the pathogenesis of UTI, as it results in decreased urine output, thereby reducing the natural flushing mechanism of the urinary tract that is responsible for clearing pathogens. Furthermore, a lack of adequate hydration may contribute to urinary stasis, thereby creating an environment more conducive to bacterial growth and infection.

These findings emphasize the necessity of considering region-specific environmental and socio-behavioral factors in the epidemiology of UTIs. Although warmer climates have historically been associated with elevated UTI prevalence due to favorable conditions for bacterial proliferation, our findings indicate that colder seasons, characterized by behavioral shifts such as increased sexual activity and reduced hydration, may also contribute to heightened UTI risk. This emphasizes the necessity for a more sophisticated approach to be taken in examining the seasonal factors that contribute to the occurrence of UTIs, particularly in regions where climatic and cultural factors may deviate from global patterns.

The findings of this study challenge the current understanding of seasonal trends in UTI occurrence, indicating that colder months may have a more pronounced impact on UTI incidence than previously assumed, particularly in specific geographical contexts such as Gabon. Further research is required to elucidate the mechanisms by which these seasonal and behavioral factors interact, in order to provide a more informed basis for the development of public health strategies for UTI prevention and management in diverse environmental settings.

Regarding the clinical categories of UTIs, cystitis and pyelonephritis at risk of complication were significantly associated with the age group ≤ 17 years. Indeed, it has been shown that pediatric UTIs are prevalent and frequently associated with a high risk of sepsis and mortality [30]. Additionally, *E. coli* isolates were linked to female UTIs, while *K. pneumoniae* was significantly associated with the age group ≤ 17 years. These findings are consistent with those of previous studies conducted in southeastern Gabon [13,14].

The results of this study highlight the importance of targeting women and young people in UTI prevention strategies. Early detection, appropriate management and effective prevention strategies may reduce the burden of UTIs in these specific populations. While our findings corroborate those of previous studies and reinforce the prevailing paradigm in Gabon, it is crucial to underscore that this trend is not universally observed on a global scale. *E. coli* is typically regarded as the primary causative agent of UTIs, even among younger populations. However, the high prevalence of *K. pneumoniae* in our study gives rise to considerable concern. The elevated prevalence of *K. pneumoniae* may signify a shift in antibiotic resistance profiles and pathogenic mechanisms within this species, thereby necessitating close observation.

This study found relatively high resistance rates of bacterial isolates to several antibiotics tested. Among *E. coli* isolates, resistance to antibiotics such as ampicillin, ticarcillin, cephalothin, nalidixic acid, trimethoprim-sulfamethoxazole, ofloxacin, and amoxicillin-clavulanic acid has raised significant concerns in both cystitis and pyelonephritis. Similar trends have been observed in *K. pneumoniae* isolates, showing resistance to antibiotics such as amoxicillin-clavulanic acid, cephalothin, cefotaxime, ceftazidime, cefepime, and trimethoprim-sulfamethoxazole. These findings agree with those if previous studies conducted in Djibouti, Cameroon, Central African Republic, Chad, and Senegal [12,18,31,32,33]. In the present study, 45% of *E. coli* and 45% of *K. pneumoniae* strains isolated from cystitis cases were multidrug-resistant to antibiotics, while a comparable resistance phenotype was observed in 38% of *E. coli* and 55% of *K. pneumoniae* strains isolated from pyelonephritis. A comparable outcome was observed at Djibouti among isolates of the *Enterobacterales* family [12].

The data pertaining to *E. coli* indicate a notable surge in antibiotic resistance across all tested drugs from 2021 to 2023. This increase is likely to be a consequence of the overuse and misuse of antibiotics, which has resulted in the emergence of multidrug-resistant strains. The increasing prevalence of antibiotic resistance underscores the imperative for the expansion of antimicrobial resistance surveillance programs in Gabon, with the objective of more effectively monitoring and addressing evolving trends. In addition, the trend underscores the importance of antimicrobial stewardship programs to promote more responsible antibiotic prescribing practices. Without immediate action, increasing resistance will compromise the effectiveness of current treatments, leading to greater challenges in managing common infections.

The emergence of high rates of resistance to commonly used antibiotics, including ampicillin, amoxicillin-clavulanic acid and trimethoprim-sulfamethoxazole, may be indicative of their inappropriate use.

It is reasonable to suggest that the significantly increased resistance to trimethoprim/sulfamethoxazole (co-trimoxazole) observed among the bacterial isolates in this study may be partially attributed to specific environmental factors present in the study area. These factors include the routine use of co-trimoxazole as a prophylactic treatment for individuals infected with the human immunodeficiency virus (HIV) and the periodic use of sulfadoxine/pyrimethamine for malaria prevention during pregnancy, as supported by recent studies conducted in Cameroon and Tunisia [18,34].

The economic impact of antibiotic resistance is considerable, largely due to the increased use of more expensive antibiotics, which in turn raises healthcare costs. Furthermore, this resistance results in additional medical expenses for patients, which significantly contribute to the phenomenon of medical poverty. The economic consequences are not solely financial; they also encompass social effects and a reduction in workforce productivity [35]. Furthermore, our findings indicate that humidity may facilitate the emergence of multidrug-resistant bacterial strains. Recent studies have demonstrated that humidity plays a significant role in the dynamics of antibiotic resistance, including the emergence of bacterial multidrug resistance [36,37]. This observation lends support to the hypothesis that elevated rates of antibiotic-resistant bacteria are associated with regions characterized by high humidity levels. This reinforces the argument that climatic conditions must be integrated into antibiotic resistance surveillance programs [38]. The influence of humidity on bacterial survival, transmission, and the potential enhancement of resistance mechanisms underscores the need for a broader, climate-conscious approach to monitoring and controlling antimicrobial resistance.

Furthermore, the conjunction of these economic and environmental factors serves to intensify the burden of antibiotic-resistant infections, not only on healthcare systems but also on the socio-economic fabric of affected populations. Consequently, the issue of antibiotic resistance must be addressed from a multifaceted perspective, taking into account not only the medical implications but also the economic, social, and environmental consequences. In the absence of a comprehensive approach, the growing threat of antimicrobial resistance will persist in undermining both public health and economic stability, particularly in vulnerable regions where environmental conditions, such as humidity, play a significant role.

## 4. Materials and Methods

### 4.1. Study Design, Geographical Scope, and Target Population

This study was carried out from January 2019 to December 2023. It included non-hospitalized patients of both genders who required a cytobacteriological examination of urine (ECBU) at the microbiology laboratory of the Interdisciplinary Center for Medical Research of Franceville (CIRMF). Franceville is the capital of the Haut-Ogooué province, with approximately 250,000 residents, and shares a border with the Republic of Congo. Only adult patients or legal guardians of minor patients who presented a verified medical prescription completed by a licensed healthcare provider were considered for inclusion in the study after giving their informed written consent. Patients were categorized into three age groups: children (0 to 17 years), adults (18 to 49 years), and seniors (50 years and older).

### 4.2. Sample and Climate Data Collection

Urine samples were collected from patients who willingly participated in the study, as previously described [9,24]. Clinical data of the patients were obtained from their medical records, while sociodemographic information was gathered through a structured questionnaire.

In order to evaluate the relationship between seasonal climatic variations and the incidence of multidrug-resistant infections caused by *E. coli* and *K. pneumoniae*, a principal component Analysis (PCA) was performed utilizing climate data from the Haut-Ogooué province. The data were obtained from the Gabonese Agency for Space Studies and Observations (AGEOS). The climatic variables included in the analysis were monthly maximum humidity (MH), monthly minimum humidity (mH), annual maximum humidity (AMH), annual minimum humidity (AmH), temperature variables (monthly maximum temperature (MT), monthly minimum temperature (mT), annual average temperature (AT)), precipitation-related factors (annual number of precipitation days (R), annual number of thunderstorm days (NSD), and annual number of rainy days (NRD)). These observations were aggregated on a monthly and annual basis over the period from January 2019 to December 2023. In Gabon, the annual distribution of seasons is as follows: a short dry period (SD) from December to February, a long rainy season (LR) from February to May, a long dry period (LD) from May to September, and a short rainy season (SR) from September to December. A principal component analysis (PCA) was conducted to reduce the dimensionality of the dataset and to identify the key factors contributing to multidrug resistance variation. The PCA was conducted on the correlation matrix of the climatic variables, with the results interpreted using the first two principal components, which collectively explained 56.47% of the total variance. PCA plots were employed to illustrate the contribution of each climatic variable to the overall variability of the data and to differentiate between multidrug-resistant and non-multidrug-resistant uropathogens.

### 4.3. Culture and Identification of Bacterial Isolates

The culture and identification of bacterial isolates were performed as previously described [9]. Briefly, the bacterial culture consisted of aseptically inoculating ten microliters (10 µL) of total urine using a sterile single-use loop in a level 2 microbiological safety station. The inoculation was carried out systematically on Agar Media, CLED (Cystine-Lactose-Electrolytes-Deficient; Biomérieux, France) and MacConkey (McC; Biomérieux, France). Urine samples were inoculated within two (2) hours of collection to prevent contamination. The inoculated media were incubated aerobically in a bacteriological incubator at 35 °C for 18 to 24 h. According to Kass criteria, the presence of ≥10^5^ colony forming units (CFU)/mL was considered positive; a colony number < 10^5^ CFU/mL or with more than two (2) types of bacterial colonies was considered to indicate contamination [39,40]. The identification of *E. coli* and *K. pneumoniae* isolates was conducted using the GN identification card of the VITEK-2 automated system (Biomérieux, Marcy-l’Etoile, France) subsequent to Gram staining and oxidase testing.

### 4.4. Antibiotic Sensitivity Test

The antibiotic sensitivity of *E. coli* and *K. pneumoniae* isolates was determined by the diffusion disk method (Kirby–Bauer) on Mueller–Hinton (MH) agar (bioMérieux, Marcy-l’Étoile, France) in accordance with the recommendations of the European Committee on Antimicrobial Susceptibility Testing that were in effect during each year of the study [41]. Briefly, MH agars were inoculated with a standardized suspension (0.5 McFarland) of each *E. coli* and *K. pneumoniae* isolate from the 24 h primary cultures. Antibiotic disks (Oxoid, Basingstoke, Hampshire, UK) were firmly placed on the surface of the seeded agar plates. The culture media were then incubated at 35 °C for 24 h. The inhibition diameters surrounding each antibiotic were then measured using a digital caliper and interpreted according to EUCAST guidelines. A total of 19 antibiotics from 11 different classes were used, including ampicillin, amoxicillin-clavulanic acid, piperacillin-tazobactam, ticarcillin, cephalothin, cefoxitin, cefotaxime, ceftazidime, cefepime, ertapenem, imipenem, gentamicin, tobramycin, amikacin, nalidixic acid, ofloxacin, ciprofloxacin, nitrofurantoin, and trimethoprim-sulfamethoxazole. Multidrug-resistance (MDR) bacteria were defined as bacteria resistant to three or more classes of antimicrobial drugs, as previously described [42]. Any isolates of *E. coli* and *K. pneumoniae* that showed resistance to one or more antibiotics in three or more antibiotic classes were categorized as having MDR. Following this classification, the percentages of MDR isolates were determined based on the total number of *E. coli* and *K. pneumoniae* isolates.

### 4.5. Categorization of Urinary Tract Infections (UTIs)

#### 4.5.1. Simple Urinary Tract Infections

Urinary tract infections were classified as simple when they occurred in patients without any risk factors for complications [3].

#### 4.5.2. Complication-Risk Urinary Tract Infections

Complication-risk urinary tract infections are those that occur in patients who present with at least one factor that may exacerbate the infection or complicate its management. These factors include clinically documented organic or functional abnormalities of the urinary tract, male gender, pregnancy, advanced age (specifically patients over 65 years with more than three frailty criteria according to Fried’s criteria, or patients over 75 years), infants under 3 months of age due to the increased risk of bacteremia, the presence of an underlying uropathy, a state of immunosuppression, or signs of severe dehydration [3].

### 4.6. Statistical Analyses

Statistical analyses were performed with R software, version 4.0.2 and SPSS version 20 (IBM, Armonk, NY, USA). The impact of sociodemographic characteristics and seasonality on the occurrence of cystitis and pyelonephritis in the study population was assessed using Pearson’s chi-square test. This test was also used to compare the prevalence of antibiotic-resistant isolates from cystitis and pyelonephritis. A principal component analysis (PCA) was conducted to ascertain the correlation between multidrug resistance to antibiotics and climatic factors, using FactoMineR and Factoextra software. PCA was chosen as a dimensionality reduction method to synthesize and minimize the loss of information from climatic factors. Logistic regression was used to assess the impact of the socio-clinical parameters of patients on the occurrence of cystitis and pyelonephritis in univariate and multivariate analyses. Odds ratios (ORs) and 95% confidence intervals are presented. Simple linear regressions were used to assess the temporal evolution of antibiotic resistance. *p*-values < 0.05 were considered statistically significant.

## 5. Conclusions

This study provides an overview of the antimicrobial resistance profiles and epidemiological factors associated with urinary tract infections caused by *E. coli* and *K. pneumoniae* in a population from southeastern Gabon. The results show alarming rates of resistance to commonly used antibiotics and highlight the clinical and public health challenges posed by these infections.

Moreover, this study underscores the critical importance of taking into account sociodemographic and climatic variables when addressing urinary tract infections.

## Figures and Tables

**Table 1:** The socio-clinical, seasonal, and temporal parameters of patients stratified by confirmed UTI cases within each grouping.

Characteristics	Number (n = 3026)	UTIs (n = 949)	Percentage (%)	*p*-Value
**Sex**				
Male	1257	356	28.3	0.002
Female	1769	593	33.5	
**Age groups**				
≤17 years	1339	458	34.2	0.003
18–49 years	1363	385	28.2	
≥50 years	324	106	33.0	
**Origin**				
Urban area	2162	703	32.5	0.03
Rural area	864	246	28.4	
**Year of data collection**				
2019	803	277	34.5	
2020	853	178	21.0	
2021	283	86	30.4	<0.0001
2022	485	192	39.6	
2023	602	216	36.0	
**Seasonality**				
Long dry season	884	271	30.6	
Short dry season	543	189	34.8	0.25
Long rainy season	755	237	31.4	
Short rainy season	844	252	29.8	

**Table 2:** Univariate and multivariate logistic regression analyses of cystitis and pyelonephritis based on sociodemographic, clinical, and temporal factors.

Characteristics	All Patient	UTIs									
		Cystitis					Pyelonephritis				
	Total Number (n = 3026)	Total Number (n = 589)	cOR (95% CI)	*p*-Value	aOR (95% CI)	*p*-Value	Total Number (n = 360)	cOR (95% CI)	*p*-Value	aOR (95% CI)	*p*-Value
**Age groups (years)**											
≤17	1339	235 (18%)	0.75 (0.56–1.02)	0.07	-	-	223 (17%)	1.65 (1.12–2.41)	0.01	1.39 (0.94–2.06)	0.09
18–49	1363	283 (21%)	0.93 (0.69–1.25)	0.64	-	-	102 (7%)	0.66 (0.44–1)	0.05	-	-
≥50 (ref)	324	71 (22%)	-	-	-	-	35 (11%)	-	-	-	-
**Sex**											
Male (ref)	1257	207 (16%)	-	-	-	-	149 (12%)	-	-	-	-
Female	1769	382 (22%)	1.39 (1.15–1.68)	<0.001	0.7 (0.58–0.85)	<0.001	211 (12%)	1 (0.80–1.25)	0.95	-	-
**Origin**											
Urban	2162	440 (20%)	1.22 (0.99–1.50)	0.05	-	-	263 (12%)	1.09 (0.85–1.4)	0.47	-	-
Rural (ref)	864	149 (17%)	-	-	-	-	97 (11%)	-	-	-	-
**Years**											
2019 (ref)	803	190 (24%)	-	-	-	-	87 (11%)	-	-	-	-
2020	853	122 (14%)	0.53 (0.41–0.69)	<0.001	0.51 (0.39–0.66)	<0.001	56 (7%)	0.57 (0.40–0.82)	<0.01	0.59 (0.41–0.84)	<0.01
2021	283	51 (18%)	0.70 (0.50–1)	0.05	-	-	35 (12%)	1.16 (0.76–1.76)	0.48	-	-
2022	485	121 (25%)	1.07 (0.82–1.39)	0.6	-	-	71 (15%)	1.41 (1.00–1.97)	0.04	1.38 (0.96–1.98)	0.08
2023	602	105 (17%)	0.68 (0.52–0.88)	0.005	0.73 (0.55–0.93)	0.02	111 (18%)	1.86 (1.37–2.5)	<0.001	1.58 (1.15–2.16)	<0.01

This table presents the results of univariate and multivariate logistic regression analyses evaluating the association between various parameters and the occurrence of cystitis and pyelonephritis. Crude and adjusted odds ratios (ORs) are reported for each variable. Crude ORs were calculated from the univariate analysis, while adjusted ORs were derived from the multivariate analysis, which controlled for the effects of all other variables included in the model. The associated *p*-values indicate the statistical significance of each association. The values in parentheses represent percentages, which indicate the proportion of patients in each category (e.g., age group, sex, origin) who had cystitis or pyelonephritis, relative to the total number of patients.

**Table 3:** Chi-square test comparison of uncomplicated and complication-risk cystitis and pyelonephritis based on sociodemographic, and clinical parameters.

Characteristics	All Infected Patient	UTIs					
		Cystitis			Pyelonephritis		
	Total Number (n = 949)	At Risk of Complication (n = 226)	Uncomplicated (n = 363)	*p*-Value	At Risk of Complication (n = 149)	Uncomplicated (n = 211)	*p*-Value
**Age groups (years)**							
**≤17**	458 (48%)	122 (54%)	113 (31%)	<0.0001	115 (77%)	108 (51%)	<0.0001
**18–49**	385 (41%)	71 (31%)	212 (58%)	<0.0001	26 (18%)	76 (36%)	<0.001
**≥50**	106 (11%)	33 (15%)	38 (11%)	0.17	8 (5%)	27 (13%)	0.03
**Origin**							
**Urban**	703 (74%)	169 (75%)	271 (75%)	1	122 (82%)	141 (67%)	<0.01
**Rural**	246 (26%)	57 (25%)	92 (25%)	1	27 (18%)	70 (33%)	<0.01
**Recurrent UTIs**							
**Yes**	116 (12%)	21 (9%)	53 (15%)	0.07	9 (6%)	33 (16%)	<0.01
**No**	833 (88%)	205 (91%)	310 (85%)	0.07	140 (94%)	178 (84%)	<0.01
**Years**							
**2019**	277 (29%)	70 (31%)	120 (33%)	0.66	43 (29%)	44 (21%)	0.10
**2020**	178 (19%)	38 (17%)	84 (23%)	0.08	17 (11%)	39 (18%)	0.09
**2021**	86 (9%)	21 (9%)	30 (8%)	0.77	13 (9%)	22 (10%)	0.72
**2022**	192 (20%)	56 (25%)	65 (18%)	0.05	36 (24%)	35 (17%)	0.1
**2023**	216 (23%)	41 (18%)	64 (18%)	0.96	40 (27%)	71 (34%)	0.2
**Uropathogens**							
***E. coli***	200 (21%)	32 (14%)	104 (29%)	<0.0001	18 (12%)	46 (22%)	0.02
***K. pneumoniae***	129 (14%)	28 (12%)	48 (13%)	0.86	24 (16%)	29 (14%)	0.63
**Other pathogens**	621 (65%)	166 (74%)	212 (58%)	<0.001	107 (72%)	136 (64%)	0.17

This table presents the results of chi-square test analyses comparing uncomplicated cystitis and cystitis at risk of complication, as well as uncomplicated pyelonephritis and pyelonephritis at risk of complication. The comparisons are based on sociodemographic, and clinical parameters. Percentages for each group are provided, along with *p*-values to assess the statistical significance of the observed differences.

## Data Availability

Data and materials supporting the conclusions of this study will be made available on request to the corresponding author.

## Supplementary Materials

The following supporting information can be downloaded at: https://www.mdpi.com/article/10.3390/antibiotics14010014/s1, Tables S1–S4: Linear Regression Analysis of Antibiotic Resistance Trends Over Five Years of *E. coli* and *K. pneumoniae* strain isolated from cystitis and pyelonephritis.

## References

[1] Yang X., Chen H., Zheng Y., Qu S., Wang H., Yi F. (2022). Disease burden and long-term trends of urinary tract infections: A worldwide report. Front. Public Health.

[2] Jodal U. (1987). The natural history of bacteriuria in childhood. Infect. Dis. Clin. N. Am..

[3] SPILF *Diagnostic et Antibiothérapie des Infections Urinaires Bactériennes Communautaires de L’adulte. Mise au Point*; Paris, France: 2015. https://www.infectiologie.com/UserFiles/File/spilf/recos/infections-urinaires-spilf-argumentaire.pdf.

[4] Akram M., Shahid M., Khan A.U. (2007). Etiology and antibiotic resistance patterns of community-acquired urinary tract infections in JNMC Hospital Aligarh, India. Ann. Clin. Microbiol. Antimicrob..

[5] Tandogdu Z., Wagenlehner F.M. (2016). Global epidemiology of urinary tract infections. Curr. Opin. Infect. Dis..

[6] Shiralizadeh S., Taghizadeh S., Asgharzadeh M., Shokouhi B., Gholizadeh P., Rahbar M., Kafil H.S. (2018). Urinary tract infections: Raising problem in developing countries. Rev. Res. Med. Microbiol..

[7] World Health Organization (2017). Prioritization of Pathogens to Guide Discovery, Research and Development of New Antibiotics for Drug-Resistant Bacterial Infections, Including Tuberculosis.

[8] Mancuso G., Midiri A., Gerace E., Marra M., Zummo S., Biondo C. (2023). Urinary tract infections: The current scenario and future prospects. Pathogens.

[9] Hu B., Ye H., Xu Y., Ni Y., Hu Y., Yu Y., Huang Z., Ma L. (2010). Clinical and economic outcomes associated with community-acquired intra-abdominal infections caused by extended spectrum beta-lactamase (ESBL) producing bacteria in China. Curr. Med. Res. Opin..

[10] Topaloglu R., Er I., Dogan B.G., Bilginer Y., Ozaltin F., Besbas N., Ozen S., Bakkaloglu A., Gur D. (2010). Risk factors in community-acquired urinary tract infections caused by ESBL-producing bacteria in children. Pediatr. Nephrol..

[11] WHO (2020). Antimicrobial Resistance.

[12] Mohamed H.S., Houmed Aboubaker M., Dumont Y., Didelot M.N., Michon A.L., Galal L., Jean-Pierre H., Godreuil S. (2022). Multidrug-resistant enterobacterales in community-acquired urinary tract infections in Djibouti, Republic of Djibouti. Antibiotics.

[13] Mouanga Ndzime Y., Onanga R., Kassa Kassa R.F., Bignoumba M., Mbehang Nguema P.P., Gafou A., Lendamba R.W., Mbombe Moghoa K., Bisseye C. (2021). Epidemiology of community origin Escherichia coli and Klebsiella pneumoniae uropathogenic strains resistant to antibiotics in Franceville, Gabon. Infect. Drug Resist..

[14] Mouanga-Ndzime Y., Onanga R., Longo-Pendy N.M., Bignoumba M., Bisseye C. (2023). Epidemiology of community origin of major multidrug-resistant ESKAPE uropathogens in a paediatric population in South-East Gabon. Antimicrob. Resist. Infect. Control..

[15] Deeny S.R., Van Kleef E., Bou-Antoun S., Hope R.J., Robotham J.V. (2015). Seasonal changes in the incidence of Escherichia coli bloodstream infection: Variation with region and place of onset. Clin. Microbiol. Infect..

[16] Eber M.R., Shardell M., Schweizer M.L., Laxminarayan R., Perencevich E.N. (2011). Seasonal and temperature-associated increases in gram-negative bacterial bloodstream infections among hospitalized patients. PLoS ONE.

[17] Schwab F., Gastmeier P., Meyer E. (2014). The warmer the weather, the more gram-negative bacteria-impact of temperature on clinical isolates in intensive care units. PLoS ONE.

[18] Nzalie R.N.-T., Gonsu H.K., Koulla-Shiro S. (2016). Bacterial Etiology and Antibiotic Resistance Profile of Community-Acquired Urinary Tract Infections in a Cameroonian City. Int. J. Microbiol..

[19] Kogan M., Ivanov S., Naboka Y. (2021). Current issues of epidemiology, etiology, risk factors and predisposing conditions of acute pyelonephritis (REVIEW-PART I). Urologiia.

[20] Alós J.I. (2005). Epidemiology and etiology of urinary tract infections in the community. Antimicrobial susceptibility of the main pathogens and clinical significance of resistance. Enfermedades Infecc. Microbiol. Clin..

[21] Singh S.K., Chandra A., Prasad A. (2020). Urinary tract infection in females, a clinicopathological correlation and appraisal. Int. J. Surg..

[22] Cortesse A., LeDuc A. (2006). Abord Clinique en Urologie.

[23] Behzadi P., Urbán E., Matuz M., Benkő R., Gajdács M. (2021). The role of gram-negative bacteria in urinary tract infections: Current concepts and therapeutic options. Adv. Microbiol. Infect. Dis. Public Health.

[24] Gonsu Kamga H., Nzengang R., Toukam M., Sando Z., Koulla Shiro S. (2014). Phénotypes de résistance des souches d’Escherichia coli responsables des infections urinaires communautaires dans la ville de Yaoundé (Cameroun). Afr. J. Pathol. Microbiol..

[25] Simmering J.E., Tang F., Cavanaugh J.E., Polgreen L.A., Polgreen P.M. (2017). The increase in hospitalizations for urinary tract infections and the associated costs in the United States, 1998–2011. Open Forum Infectious Diseases.

[26] Czaja C.A., Scholes D., Hooton T.M., Stamm W.E. (2007). Population-based epidemiologic analysis of acute pyelonephritis. Clin. Infect. Dis..

[27] Stamm W.E., McKevitt M., Roberts P.L., White N.J. (1991). Natural history of recurrent urinary tract infections in women. Rev. Infect. Dis..

[28] McDonald L.C., Banerjee S.N., Jarvis W.R., National Nosocomial Infections Surveillance System (1999). Seasonal variation of Acinetobacter infections: 1987–1996. Clin. Infect. Dis..

[29] Perencevich E.N., McGregor J.C., Shardell M., Furuno J.P., Harris A.D., Morris J.G., Fisman D.N., Johnson J.A. (2008). Summer peaks in the incidences of gram-negative bacterial infection among hospitalized patients. Infect. Control. Hosp. Epidemiol..

[30] Morris B.J., Wiswell T.E. (2013). Circumcision and lifetime risk of urinary tract infection: A systematic review and meta-analysis. J. Urol..

[31] Hima-Lerible H., Ménard D., Talarmin A. (2003). Antimicrobial resistance among uropathogens that cause community-acquired urinary tract infections in Bangui, Central African Republic. J. Antimicrob. Chemother..

[32] Yandai F.H., Ndoutamia G., Nadlaou B., Barro N. (2019). Prevalence and resistance profile of Escherichia coli and Klebsiella pneumoniae isolated from urinary tract infections in N’Djamena, Tchad. Int. J. Biol. Chem. Sci..

[33] Dia M., Chabouny H., Diagne R. (2015). Profil antibiotypique des bactéries uropathogènes isolées au CHU de Dakar. Rev. Afr. D’urologie D’andrologie.

[34] Smaoui S., Abdelhedi K., Marouane C., Kammoun S., Messadi-Akrout F. (2015). Résistance aux antibiotiques des entérobactéries responsables d’infections urinaires communautaires à Sfax (Tunisie). Médecine Mal. Infect..

[35] Ahmad M., Khan A.U. (2019). Global economic impact of antibiotic resistance: A review. J. Glob. Antimicrob. Resist..

[36] Beizman-Magen Y., Orevi T., Kashtan N. (2024). Hydration conditions as a critical factor in antibiotic-mediated bacterial competition outcomes. bioRxiv.

[37] Shawver S., Ishii S., Strickland M.S., Badgley B. (2024). Soil type and moisture content alter soil microbial responses to manure from cattle administered antibiotics. Environ. Sci. Pollut. Res..

[38] MacFadden D.R., McGough S.F., Fisman D., Santillana M., Brownstein J.S. (2018). Antibiotic resistance increases with local temperature. Nat. Clim. Chang..

[39] Kass E.H. (1957). Bacteriuria and the diagnosis of infections of the urinary tract: With observations on the use of methionine as a urinary antiseptic. AMA Arch. Intern. Med..

[40] Kass E.H. (2002). Asymptomatic infections of the urinary tract. J. Urol..

[41] The European Committee on Antibiotic Susceptibility Testing Breakpoint Tables for Interpretation of MICs and Zone Diameters. http://www.eucast.org/clinical_breakpoints/.

[42] Magiorakos A.P., Srinivasan A., Carey R.B., Carmeli Y., Falagas M.E., Giske C.G., Harbarth S., Hindler J.F., Kahlmeter G., Olsson-Liljequist B. (2012). Multidrug-resistant, extensively drug-resistant and pandrug-resistant bacteria: An international expert proposal for interim standard definitions for acquired resistance. Clin. Microbiol. Infect..

