# Supplementary material for: Trends in Escherichia coli and Klebsiella pneumoniae Urinary Tract Infections and Antibiotic Resistance over a 5-Year Period in Southeastern Gabon
Source: Antibiotics (Basel). 2024 Dec 28;14(1):14. doi: 10.3390/antibiotics14010014 (PMC11762395; doi:10.3390/antibiotics14010014)
Supplement: Supplementary file 1 [file antibiotics-14-00014-s001.zip › antibiotics-3218020-supplementary.pdf]

Table S1 Linear regression analysis of antibiotic resistance trends over five years of *E. coli* strains isolated from patients with cystitis.

**Antibiotic**	**Estimate**	**Std. Error**	**t-value**	**p-value**
Ampicillin	6.10	5.87	1.04	0.37
Amoxicillin-clavulanic acid	3.10	4.61	0.673	0.54
Cefotaxime	8.90	7.74	1.15	0.33
Gentamicin	3.60	6.98	0.516	0.64
Ciprofloxacin	4.70	4.75	0.989	0.39
Nitrofurantoin	7.40	2.17	3.41	**0.04**

This table presents the results of simple linear regressions for each antibiotic, analyzing the trend of antibiotic resistance over a five-year period. The table includes estimates, standard errors, t-values, p-values for each antibiotic.

Table S2 Linear regression to assess the effect of year on resistance to different antibiotics of *E. coli* strains isolated from pyelonephritis.

**Antibiotic**	**Estimate**	**Std. Error**	**t-value**	**p-value**
Ampicillin	-6.50	2.71	-2.40	0.09
Amoxicillin-clavulanic acid	-8.30	5.05	-1.64	0.19
Cefotaxime	1.80	8.03	0.224	0.83
Gentamicin	-1.70	9.79	-0.174	0.87
Ciprofloxacin	-5.30	10.3	-0.512	0.64
Nitrofurantoin	2.60	1.50	1.73	0.18

This table presents the results of simple linear regressions for each antibiotic, analyzing the trend of antibiotic resistance over a five-year period. The table includes estimates, standard errors, t-values, p-values for each antibiotic.

Table S3 Linear regression to assess the effect of year on resistance to different antibiotics of *K. pneumoniae* strains isolated from cystitis.

**Antibiotic**	**Estimate**	**Std. Error**	**t-value**	**p-value**
Amoxicillin-clavulanic acid	-7.50	6.89	-1.09	0.35
Cefotaxime	-7.20	6.46	-1.11	0.34
Gentamicin	-3.00	4.67	-0.642	0.56
Ciprofloxacin	0.5	2.06	0.243	0.82
Nitrofurantoin	0.2	1.27	0.157	0.88

This table presents the results of simple linear regressions for each antibiotic, analyzing the trend of antibiotic resistance over a five-year period. The table includes estimates, standard errors, t-values, p-values for each antibiotic.

Table S4 Linear regression to assess the effect of year on resistance to different antibiotics of *K. pneumoniae* strains isolated from pyelonephritis.

**Antibiotic**	**Estimate**	**Std. Error**	**t-value**	**p-value**
Amoxicillin-clavulanic acid	-9.50	8.09	-1.17	0.32
Cefotaxime	-3.90	11.3	-0.346	0.75
Gentamicin	0.100	10.1	0.009	0.99
Ciprofloxacin	1.00	6.11	0.164	0.88
Nitrofurantoin	11.6	5.37	2.16	0.12

This table presents the results of simple linear regressions for each antibiotic, analyzing the trend of antibiotic resistance over a five-year period. The table includes estimates, standard errors, t-values, p-values for each antibiotic.
